# The Role of Nursing Care in the Management of Patients with Traumatic Subarachnoid Hemorrhage

**DOI:** 10.31661/gmj.v12i.3013

**Published:** 2023-08-20

**Authors:** Qun Miao, Yan Yan, Mengjie Zhou, Xueqi Sun

**Affiliations:** ^1^ Department of Neurosurgery, Funan County People’s Hospital, Fuyang, Anhui 236300, China

**Keywords:** Traumatic Subarachnoid Hemorrhage, Nursing Care, Brain Injuries, Intracranial Pressure; Emotional Support

## Abstract

Traumatic subarachnoid hemorrhage (tSAH) is a critical condition that requires comprehensive management to optimize patient outcomes. Nursing care plays a key role in the overall management of patients with tSAH via various aspects of care, including neurological assessment, monitoring, intervention, and education. In this review, we aim to evaluate the significant contributions of nursing care in managing patients with tSAH. Nurses perform initial neurological assessments, including the glasgow coma scale, pupil reactivity, vital signs, and sensory-motor evaluations. These assessments provide valuable information for early identification of deteriorating neurological status and prompt intervention. Additionally, nurses closely monitor intracranial pressure (ICP), cerebral perfusion pressure, and other hemodynamic parameters, assisting in the prevention and timely detection of secondary brain injury. For example, some strategies to manage ICP include elevating the head of the bed, maintaining adequate oxygenation and ventilation, administering proper medications, and ensuring fluid and electrolyte balance. Also, through careful monitoring, early recognition, and appropriate preventive measures, nursing care could prevent complications, including infections, deep vein thrombosis, and pressure ulcers. Furthermore, nursing care extends beyond physical management and encompasses psychosocial support for patients and their families. Nurses establish therapeutic relationships, providing emotional support, education, and counseling to alleviate anxiety, address concerns, and facilitate coping mechanisms. Education regarding medication management, lifestyle modifications, and the importance of regular follow-up enhances patient compliance and promotes long-term recovery.

## Introduction

Traumatic subarachnoid hemorrhage (tSAH) is a critical condition characterized by bleeding into the subarachnoid space following a traumatic brain injury [1]. It poses a significant challenge for healthcare professionals, particularly nurses, which play a crucial role in managing patients with tSAH [2]. The complex nature of tSAH demands a comprehensive nursing care approach that includes assessment, intervention, education, and ongoing support [3][4]. Hence, this study aimed to short review the valuable role of nursing care in managing patients with tSAH, highlighting the essential responsibilities of nurses and their impact on patient outcomes.

1. Initial Assessment and Monitoring

Nurses play a key role in continuously assessing and monitoring the patient’s neurological status, vital signs, and level of consciousness [5]. During the initial assessment, the nurse should collect important information about the patient’s medical history, including any previous head injuries, current medications, and allergies [6].

Also, they should assess the severity of the trauma and document the Glasgow Coma Scale (GCS) score, which helps determine the level of consciousness and neurological functioning [7]. Additionally, vital signs such as blood pressure (BP), heart rate, and respiratory rate should be monitored continuously to detect any signs of deterioration. By closely monitoring these parameters, nurses can provide timely and effective care, optimize patient outcomes, and contribute to the overall management of tSAH patients [8].

1.1. Neurological Examinations

Neurological assessment is a fundamental aspect of caring for patients with tSAH. The nurse should closely monitor the patient’s GCS, pupillary response, motor strength, and sensation [9]. Any changes in these parameters may indicate worsening intracranial pressure (ICP) or neurological deterioration [10]. Frequent examinations of cranial nerve function, including facial symmetry, eye movements, and gag reflex are important to identify any deficits that may require immediate intervention [11].

1.2. Monitoring Vital Signs

Monitoring vital signs and oxygenation status is crucial in managing tSAH. Close attention should be given to BP control to prevent rebleeding and/or exacerbation of cerebral edema [12]. However, hypotension should be avoided, as it can lead to cerebral hypoperfusion; hence, careful management of BP to maintain adequate cerebral perfusion pressure is necessary [13]. Also, oxygen saturation levels should be monitored continuously, and supplemental oxygen should be administered as needed to ensure optimal tissue oxygenation [14].

1.3. Laboratory and Imaging Studies

Laboratory monitoring is also an integral part of nursing care for patients with tSAH. Serial blood tests, including complete blood count, coagulation profile, electrolytes, and arterial blood gases, are necessary to evaluate the patient’s overall condition, detect any abnormalities, and guide appropriate interventions [15]. Serial imaging studies such as computed tomography (CT) scans and/or magnetic resonance imaging (MRI) may be performed to assess the extent of bleeding, identify underlying structural damage, or monitor treatment response [16][17].

2. Interventions

2.1. Medication Administration

In some patients, medications are administered to manage symptoms, prevent complications, and promote recovery [18]. One of the primary medications commonly administered is analgesic [19]. tSAH can cause severe headaches and discomfort, and nurses must carefully assess and administer appropriate analgesics to alleviate the patient’s pain [20].

In addition to pain management, nurses administer medications to prevent seizures [21]. Indeed, tSAH can increase the risk of seizures, and antiepileptic drugs are commonly prescribed to reduce this risk [22]. Nurses need sufficient knowledge regarding the specific antiepileptic medications used and their dosage requirements to ensure that patients receive the correct treatment [23]. Furthermore, medications to manage BP may be necessary for patients with tSAH. Indeed, elevated BP could worsen the condition and increase the risk of rebleeding [24]. Hence, nurses closely monitor the patient’s BP and administer antihypertensive medications as prescribed by the healthcare provider to maintain stable BP levels [25]. Another important aspect of medication administration for patients with tSAH is preventing thromboembolic events [26]. These patients are at an increased risk of developing thrombosis, which can lead to stroke or other serious complications [27]. Nurses administer prophylactic anticoagulant medications such as low molecular weight heparin or fondaparinux to reduce the risk of thrombus formation while closely monitoring the patient for adverse effects [28]. Overall, medication administration for patients with tSAH requires nurses with the high level of skill as well as knowledge [29]. In other words, they should be familiar with proper drug dosages, potential side effects, drug interactions, and contraindications [30]. Additionally, nurses must adhere to medication administration protocols, double-check dosages, and ensure accurate documentation to maintain patient safety [31]. Hence, by effectively managing pain, preventing seizures, controlling BP, and minimizing thromboembolic events, nurses contribute significantly to the overall care and recovery of patients with tSAH.

2.2. ICP Monitoring

In the management of patients with tSAH, ICP monitoring plays a crucial role, and nurses play a significant part in its implementation and management [32]. ICP monitoring involves measuring the pressure within the skull to assess the status of brain tissue perfusion and detect any abnormalities that could lead to further complications [33]. Nurses are responsible for setting up and maintaining the ICP monitoring system. They ensure the equipment is correctly calibrated, properly placed, and connected to the patient’s intraventricular or intraparenchymal catheter [34]. This process may require precision and attention to detail to avoid potential errors that could compromise the accuracy of ICP measurements.

Regular monitoring and interpretation of ICP values are also essential nursing responsibilities [35]. Nurses frequently assess and document ICP readings, closely monitoring any fluctuations or abnormal trends [36]. By closely monitoring ICP levels, nurses could promptly identify and report any concerning changes to the healthcare team, enabling timely interventions to prevent secondary brain injuries.

Indeed, nurses are involved in implementing strategies to manage ICP within acceptable limits. They collaborate with other healthcare professionals, such as neurosurgeons and intensivists, to develop and execute individualized care plans [37]. These plans may include elevating the head of the bed, maintaining adequate cerebral perfusion pressure, administering medications to control ICP, and managing ventilation parameters to optimize oxygenation and carbon dioxide levels [38][39].

Furthermore, nurses provide ongoing education and support to patients and their families regarding the purpose and importance of ICP monitoring [40], which provides the rationale behind these interventions.

2.3. Fluid and Electrolyte Balance

Regarding various complications of tSAH, including cerebral edema and electrolyte imbalances, close monitoring and appropriate interventions to provide both fluid and electrolyte balance are essential for optimal patient outcomes [41]. Maintaining euvolemia prevents complications such as hypovolemia or hypervolemia [42]. Also, adequate hydration is necessary to optimize cerebral perfusion and prevent secondary brain injury. However, excessive fluid administration could contribute to cerebral edema and increased ICP [43]. Nurses should closely monitor vital signs, neurological status, urine output, and laboratory values to evaluate fluid levels accurately [44][45]. They may collaborate with other healthcare team members, e.g., pharmacists, to determine appropriate fluid replacement strategies, which may include crystalloids or colloids [46].

Electrolyte balance is another critical aspect of patient care in tSAH. Electrolytes such as sodium, potassium, calcium, and magnesium play vital roles in cellular function, nerve conduction, and maintaining osmotic pressure [47]. tSAH can disrupt electrolyte balance due to various factors, including blood loss, diuretic therapy, or inadequate intake [48]. Hence, nurses must regularly monitor serum electrolyte levels and promptly intervene to correct any imbalances. For example, hyponatremia, or hypernatremia, can impact cerebral function and worsen patient outcomes [49]. So, nurses should administer electrolyte replacements or collaborate with the healthcare team to adjust medication dosages to restore and maintain electrolyte balance. In addition to monitoring fluid and electrolyte balance, nurses should consider factors such as renal function and urine output [50]. Maintaining adequate renal perfusion is crucial to prevent acute kidney injury, which can further complicate the patient’s condition [51]. By closely monitoring urine output and assessing renal function through laboratory tests, nurses can identify early signs of impaired renal function and implement appropriate interventions, including adjusting fluid administration, using diuretic medications, or collaborating with the healthcare team to address underlying causes [52].

2.4. Preventing Infections

By following specific guidelines and employing evidence-based practices, nurses can effectively reduce the incidence of infections in patients with tSAH [53].

Firstly, meticulous hand hygiene is necessary for preventing infections. Nurses should adhere to strict handwashing protocols, utilizing soap and water or alcohol-based sanitizers before and after any direct contact with patients [54]. This practice significantly reduces the transmission of pathogens and protects both the patient and the healthcare provider from potential infections [55].

Another vital strategy is to maintain a clean and sterile environment. Regular cleaning and disinfection of patient care areas, such as bedside tables, equipment, and surfaces, are essential [56]. Nurses should ensure that all equipment used for invasive procedures, such as catheters or drains, are appropriately sterilized and handled using aseptic techniques [57].

Also, implementing proper wound care techniques is crucial in preventing surgical site infections in patients with tSAH [58]. Indeed, nurses should meticulously assess and monitor the integrity of surgical incisions or any open wounds. Regular dressing changes using sterile techniques and appropriate antimicrobial agents help reduce the risk of infection [59][60]. Additionally, closely monitoring for signs of infection, such as redness, swelling, or drainage, allows for early detection and intervention [57]. In addition, effective management of invasive devices, including urinary catheters and central venous lines, is also vital in infection prevention [61]. Nurses should follow strict protocols for their insertion, maintenance, and removal [61][62]. Regular monitoring for any signs of infection or complications related to these devices is crucial [61]. Indeed, early identification and prompt intervention can help prevent the spread of infection and subsequent complications [63].

Education and communication are important aspects of infection prevention. Nurses should educate patients and their families about the importance of infection control measures, including hand hygiene, wound care, and recognition of signs and symptoms of infection [64][65]. Additionally, clear communication among the healthcare team regarding isolation precautions, patient status, and any specific infection risks is critical to ensure a cohesive approach [66].

2.5. Emotional Support

Managing patients with tSAH requires a comprehensive approach that addresses both the physical and emotional well-being of the individual. Emotional support strategies play a key role in helping patients cope with the psychological impact of their condition [67].

Remember, each patient’s experience with tSAH is unique, and their emotional needs may differ. Tailoring emotional support strategies to individual preferences and circumstances is essential for effective management [68]. Collaborating with a multidisciplinary team that includes healthcare professionals, psychologists, and social workers can provide comprehensive care and support for patients with tSAH [67].

2.5.1. Provide Clear and Honest Communication

Establishing open and transparent communication with patients is essential. It is important to explain the diagnosis, treatment options, and potential outcomes in a clear and compassionate manner [68]. Offering reassurance and answering any questions and/or concerns could help reduce anxiety and foster trust [69].

2.5.2. Empathetic Listening and Validation

Patients with tSAH often experience various emotions, such as fear, confusion, frustration, and grief [70][71]. Active listening and validating their feelings can make them feel understood and supported. Encouraging them to express their emotions and offering empathy can help them process their trauma and adjust to their new reality [72].

2.5.3. Psychosocial Interventions

Engaging patients in psychosocial interventions, e.g., counseling, support groups, and therapy sessions, can be immensely beneficial [73]. These interventions provide a safe space for patients to share their experiences, learn coping strategies, and receive guidance from professionals who specialize in trauma-related care [74].

2.5.4. Education and Information

Providing educational resources about tSAH, its management, and recovery can encourage patients to participate actively in their care [75]. Knowledge about their condition can reduce anxiety, promote self-care, and enhance their understanding of the healing process [76][77].

2.5.5. Encourage Social Support

Building a network of social support is crucial for patients with tSAH. Social support can provide emotional stability, practical assistance, and a sense of belonging, positively impacting the patient’s overall well-being [78][79].

2.5.6. Addressing Post-Traumatic Stress

Some patients may develop post-traumatic stress disorder (PTSD) following tSAH [80]. Recognizing the signs and symptoms of PTSD, such as flashbacks, nightmares, and avoidance behaviors, is essential [81]. Referring patients to mental health professionals who are specialized in trauma can help them receive appropriate treatment and support.

2.5.7. Holistic Approaches

Incorporating holistic approaches, such as relaxation techniques, mindfulness exercises, and complementary therapies, such as art and/or music therapy, can contribute to the emotional well-being of patients [82][83]. These practices promote stress reduction, emotional regulation, and overall psychological resilience [83].

3. Rehabilitation Assistance

Early mobilization is a key strategy in rehabilitation assistance for tSAH patients [84]. Nurses work closely with physical therapists to ensure that patients start engaging in mobility exercises as soon as their medical condition allows [85]. This may involve helping the patient sit up, stand, or take their first steps. Early mobilization not only helps prevent complications such as muscle atrophy and joint stiffness but also promotes neuroplasticity and improves overall functional outcomes [86].

Communication assistance is another vital aspect of rehabilitation for tSAH patients. Many individuals with tSAH experience communication difficulties due to damage to the brain’s language centers [87]. Nurses can collaborate with speech-language pathologists to implement strategies such as augmentative and alternative communication techniques, including using picture boards or electronic devices to facilitate effective communication [88]. Additionally, nurses can provide emotional support and patience during the patient’s rehabilitation process, as frustration and anxiety may arise from the challenges of verbal expression [89].

Cognitive rehabilitation is also an essential component of managing tSAH patients. Nurses, alongside occupational therapists and neuropsychologists, can develop individualized cognitive training programs to improve attention, memory, problem-solving skills, and other cognitive functions [90][91]. These programs may include puzzles, memory games, and computer-based exercises [92]. Nurses play a critical role in guiding and supporting patients throughout these sessions, monitoring their progress, and providing feedback to optimize outcomes. Also, psychosocial support is equally important in the rehabilitation of tSAH patients [93]. Nurses can assess and address the patient’s emotional well-being, providing counseling and education to the patient and their families regarding the psychological consequences of tSAH. Also, they can facilitate support groups or connect patients with community resources to promote a sense of belonging and reduce feelings of isolation [94]. Additionally, nurses can collaborate with social workers to help address any financial or logistical challenges impeding the patient’s rehabilitation progress [95].

4. Health Education and Discharge Planning

Health education begins immediately upon admission and continues throughout the patient’s hospital stay [96]. Nurses aim to provide comprehensive information about tSAH, including its causes, risk factors, symptoms, and potential complications [97]. They explain the diagnostic tests, such as CT scans and cerebral angiography, and help patients understand their results. By promoting health literacy, nurses encourage patients to actively participate in their care and make informed decisions [98].

Furthermore, nurses educate patients about the treatment options available for tSAH. This may include surgical interventions, as well as medical management strategies to control BP, prevent vasospasm, and manage pain [99][100]. They explain the purpose, benefits, and potential risks or side effects associated with each intervention, ensuring patients clearly understand their treatment plan.

Discharge planning is an integral part of managing patients with tSAH, as it involves coordinating services and resources to support the transition from the hospital to home or another healthcare setting [101]. Nurses collaborate with interdisciplinary teams, including physicians, physical therapists, occupational therapists, and social workers, to develop a comprehensive discharge plan tailored to the patient’s individual needs [102]. During discharge planning, nurses assess the patient’s functional abilities, cognitive status, and psychosocial factors that may impact their recovery. Also, they provide guidance on medication management, wound care, activity restrictions, and lifestyle modifications to prevent recurrent bleeding or complications [103][104].

In addition to physical care, nurses emphasize the importance of emotional support and coping strategies for patients and their families. They provide resources for counseling services or support groups to help individuals navigate the emotional impact of tSAH and its potential long-term effects [105].

## Conclusion

Optimal nursing care plays a vital role in the management of patients with tSAH. Nurses contribute significantly to achieving positive patient outcomes through early recognition, comprehensive assessment, vigilant monitoring, implementation of therapeutic interventions, psychosocial support, patient education, and collaborative care. Ongoing research and evidence-based practice guidelines continue to shape nursing care strategies, leading to further improvements in managing patients with tSAH and their overall quality of life.

## Conflict of Interest

The authors declare no conflict of interest.
